# Integrated Bioinformatics Analysis for the Screening of Hub Genes and Therapeutic Drugs in Androgen Receptor-Positive TNBC

**DOI:** 10.1155/2022/4964793

**Published:** 2022-09-14

**Authors:** Qiaonan Guo, Pengjun Qiu, Qingzhi Yao, Jianpeng Chen, Jianqing Lin

**Affiliations:** Thyroid & Breast Surgery, The Second Affiliated Hospital of Fujian Medical University, 950 Donghai Street, Quanzhou, China

## Abstract

As the most invasive and lethal subtype of breast cancer (BC), triple-negative breast carcinoma (TNBC) is of increasing interest. However, the androgen receptor (AR) still has an unclear role in TNBC. The current study is aimed at testing the diagnostic and therapeutic performance of novel biomarkers for AR-positive TNBC. The GSE76124 dataset was analyzed by combining WGCNA and other bioinformatics methods. Subsequently, function enrichment analysis was applied to identify the relationships between these differential expression genes (DEGs). Subsequently, the protein-protein interaction network was established, and the hub genes were identified by Cytoscape software. Eventually, the miRNA-hub gene modulate network was developed and the Drug-Gene Interaction Database (DGIdb) was applied to verify the potential drugs for AR-positive TNBC. In the current research, 88 DEGs in total were selected from the intersection of the purple module genes identified by WGCNA and limma package. TFF1, FOXA1, ESR1, AGR2, TFF3, AGR3, GATA3, XBP1, SPDEF, and TOX3 were selected as hub genes by the MCC method, which were all upregulated. The survival analysis suggested that TFF1 was the only one related to significant lower survival rate in TNBC. Ultimately, hsa-miR-520g-3p and hsa-miR-520h were found taking part in the regulation of TFF1, and 2 small molecules were identified as the potential targets for AR-positive TNBC treatment. As a result, our study suggested that hsa-miR-520g-3p, hsa-miR-520h, and TFF1 might have significant potential values for AR-positive TNBC diagnosis and prognosis prediction. TFF1, hsa-miR-520g-3, and hsa-miR-520h may serve as the novel therapeutic targets, and our findings offer further insights into the therapy of AR-positive TNBC.

## 1. Introduction

The incidence of BC is the highest of all female malignancies worldwide. In 2020, US statistics indicated that BC accounted for 30% of female malignancies and had a 15% mortality rate [1]. At present, the prognosis of breast cancer was improved by several clinical treatment methods, mainly including chemotherapy, radiation therapy, surgery, immunotherapy, and targeted therapy [2]. Although surgery is still the mainstay of early BC treatment, chemotherapy and radiotherapy are important in reducing recurrence and improving prognosis. Recently, more and more drugs targeting HER2 have been developed and identified to improve the prognosis of HER2-positive BC patents. As for the hormone receptor- (HR-) positive BC, in addition to the classic aromatase inhibitors and estrogen receptor antagonists, CDK4/6 inhibitors are used extensively in clinical practice in recent years [3]. Regrettably, the lack of biomarkers for early detection and identified targets for treatment meant that patients with TNBC were diagnosed late and benefited little from targeted or hormonal treatments [4]. Consequently, TNBC patients generally faced high risks of metastasis as well as recurrence and had a worse prognosis, with reduced overall survival (OS) and disease-free survival (DFS) [5–7]. Notably, a number of gene mutations have been previously described over the years as being highly significantly associated with an increased risk of BC. In particular, breast cancer 1 (BRCA1) and BRCA2 are tumor suppressor genes with high penetrance. They are identified to take part in the processes of activating the cell-cycle checkpoints and DNA repair to further respond to DNA damage. Consequently, the application of poly[ADP-ribose] polymerase (PARP) inhibitors targeted to PARP proteins associated with DNA repair mechanisms is shown to be efficient in BC with BRCA1/2 gene mutation [8]. Additionally, the circulating tumor DNA (ctDNA) was reported to be useful in the diagnosis and surveillance of BC. ctDNA is detected by “liquid biopsies,” which are noninvasive means by simply obtaining blood instead of tumor tissue biopsies. To date, a number of ctDNA biomarkers have been identified and used for the diagnosis and prognosis of BC, including tumor protein p53 (TP53), AKT1, and phosphatidylinositol-4,5-bisphosphate 3-kinase catalytic subunit alpha (PIK3CA) etc., but they still lack specificity for TNBC [9]. The molecular subtyping of TNBC is important for the correct classification of cancer lesions and for predicting patient prognosis. Therefore, an increasing amount of research is focused on identifying new molecules critical to TNBC in order to provide improved diagnosis, prognostic prediction, and treatment strategies for this malignant tumor.

Breast carcinoma is classified into four subtypes based on the expression of genes and receptor proteins [10]. TNBC accounts for 15-20% of all subtypes of BC and is usually described as ER-negative, PR-negative, or HER2-negative [11]. In 2011, Lehmann et al. performed a cluster analysis on the gene expression profiles of 587 patients with TNBC from 21 public databases and proposed that TNBC could be divided into 6 subtypes based on the gene expression level. They were named basal-like-1 (BL-1), basal-like-2 (BL-2), mesenchymal (M), immunomodulatory (IM), mesenchymal stem-like (MSL), and luminal androgen receptor (LAR) [12]. In 2015, Burstein et al. conducted the genome-wide analysis of 198 TNBC samples to determine 4 TNBC subtypes: basal-like immune-suppressed type (BLIS), basal-like immune-activated (BLIA), mesenchymal (MES), and LAR. At the same time, they pointed out that the prognosis of BLIS-type TNBC was poor, while BLIA-type TNBC had a good prognosis (*P* < 0.05) [13]. AR was identified as a steroid receptor superfamily member and was expressed in over 70% of BC as well as approximately 25%-35% of TNBC [14–19]. Some studies showed that AR-positive patients were notably related to a low risk of cancer recurrence and patient mortality. Nevertheless, in other studies, positive AR expression of TNBC patients was related to poorer clinical performance, and therapeutic AR blockade was worth considering as a possible endocrine therapy [14, 20–23]. Consequently, the potential therapeutic strategies for AR-positive TNBC may be provided by identification of novel biomolecules and signaling pathways.

Some oncological academics suggested that diagnostic, prognostic, and predictive values were generally essential for good biomarkers. In addition, the application of bioinformatics approaches to integrate biomarker data will provide us with new insights in biological pathways and regulatory mechanisms with disorders. Hong and colleagues used systemic and comprehensive bioinformatics to identify an 8-miRNA signature that can improve the current TNM staging system and provide a molecular assay to forecast recurrence in TNBC patients after surgery [10]. Moreover, the study conducted by Burstein et al. found novel subtype-specific targets that could be targeted for the effective treatment of TNBCs through RNA and DNA profiling analysis for the datasets from the GEO database and Baylor College of Medicine [13]. Besides, Candido et al. revealed the roles that IL6, IL6R, and IL6ST played in epigenetic regulations in cancer by use of cancer genomic and epigenomic datasets from TCGA [24]. The bioinformatics and computational analysis were applied not only in the oncology field but also in other diseases. Giambò and colleagues identified the vital genetic and epigenetic alterations related to pesticide exposure by a series of computational analyses of gene expression, miRNA expression, and DNA methylation datasets from the GEO database [25].

Our study was aimed at identifying novel effective biomarkers for TNBC especially for AR-positive TNBC. For this purpose, a series of continuous bioinformatics methods and computational statistical analysis are applied to miRNA profiling from public database.

WGCNA (weighted gene coexpression network analysis) was a systematic biological method to identify the modules of highly associated genes to establish a coexpression network on the basis of gene expression data [26]. Genes were expressed and functionally similar in the same coexpression module.

The total expression of genes in the coexpression was reflected by the first principal component, named module vector [27]. The WGCNA was employed to define the hub genes of the cluster, which could play as the potential biomarkers of disease or targets for therapy. Moreover, the molecular mechanism of BC development was able to be illustrated through the modulatory networks of the genes involved [28, 29]. WGCNA had been adopted to find several potential biomarkers in different fields, including neurodegenerative diseases, cancers, and immune disease [30, 31].

In this study, microarray data of the GSE76124 dataset was collected from the Gene Expression Omnibus database (GEO database, https://www.ncbi.nlm.nih.gov/geo/). These samples were defined as the AR-positive subtype TNBC group and the other-three-subtype (MES, BLIA, and BLIS) TNBC group [13]. WGCNA was used to establish coexpression networks for both groups, identify modules associated with AR positivity, and obtain the key genes in the modules. Subsequently, the limma package was applied to recognize the differentially expressed genes (DEGs) between the AR-positive subtype and the other three subtypes of TNBC tissues. The candidate genes related to AR-positive TNBC were finally selected by combining DEGs and WGCNA algorithms. Kyoto Encyclopedia of Genes and Genomes (KEGG) pathway analysis and Gene Ontology (GO) analysis were conducted to elucidate the possible signaling pathways and biological functions of the DEGs. The protein-protein interaction (PPI) network was established via the Search Tool for the Retrieval of Interacting Genes (STRING, https://string-db.org/, version 11.0) database and visualized by Cystoscape software. Subsequently, the topological analysis methods were used to screen out the hub genes. The expression of each hub gene between normal tissue and TNBC tissue was verified through Gene Expression Profiling Interactive Analysis (GEPIA, http://gepia.cancer-pku.cn/) (*P* < 0.05). The online database Kaplan–Meier plotter (KM plotter, http://kmplot.com/analysis/) was adopted to evaluate the prognostic value of the hub genes. After that, TFF1 was considered as a crucial gene of AR-positive TNBC. Furthermore, the target miRNAs of TFF1 were screened out by means of the intersection of the Encyclopedia of RNA Interactomes (ENCORI, http://starbase.sysu.edu.cn/) and TargetScan (http://www.targetscan.org/vert_72/; version 7.2), and the correlation between TFF1 and RNA expression was verified. After that, the regulatory miRNAs of TFF1 were identified, has-miR-520g-3p and hsa-miR-520h, which were considered to be associated with the regulatory mechanism of AR-positive TNBC development. Eventually, the miRNA-hub gene network was further established. The Drug-Gene Interaction Database (DGIdb) was utilized to verify and find the candidate drugs for AR-positive TNBC. Therefore, our work was aimed at illustrating the potential molecularly mechanisms in promoting the prognosis of AR-positive TNBC. The results may further provide insights into the diagnosis and therapies of AR-positive TNBC.

## 2. Materials and Methods

### 2.1. Public Datasets and Data Preprocessing

The gene expression microarray datasets (GSE76124 and GSE167213) were retrieved from GEO, which were processed on the GPL570 platform (Affymetrix Human Genome U133 Plus 2.0 Array). Inclusion criteria were as follows: (a) mRNA expression data were available; (b) more than 100 TNBC samples were available with complete clinical information; (c) the AR state for each sample was exact. The dataset GSE76124 contained 198 TNBC samples and provided information about TNM classification and molecular subtypes of TNBC. 37 LAR (AR positive) samples, 47 MES (mesenchymal) samples, 60 BLS (basal-like-1/2) samples, and 54 BLIA (basal-like immune-activated) samples were included in GSE76124. The clinical and molecular features of the 198 TNBC samples are shown in supplemental table 1 [13]. The FITPLM function in the AFFYPLM package was employed to conduct the regression calculation to further assess the dataset. Subsequently, the quality of the dataset was evaluated by drawing a weight map, relative logarithmic expression map, residual symbol map, and RNA degradation map. Besides, the KNN method was adopted to add the missing values [32]. Probes with gene annotation and matched only one genetic symbol were included in the current study. Eventually, 23,519 genes in 198 samples from the GEO database were screened out for the coexpression network establishment after ranking the variance of the descending alignments.

### 2.2. Weighted Gene Coexpression Network Analysis (WGCNA)

As an approach for gene set expression analysis, WGCNA was adopted to establish a network, in which the genes and the interrelationships between genes were represented as the points and lines, respectively. The coexpression network was established by use of the R package WGCNA (http://www.r-project.org/) in the R environment [33]. Generally, matrices of paired Pearson correlation coefficients were created to assess the similarities between genes in TNBC patients. Subsequently, the power adjacency function was applied to realize the conversion of the similarity matrix and the adjacency matrix. According to the scale-free network, we further built the topology of the coexpression network. And the function of soft connectivity from the WGCNA package was employed to select the soft-threshold power *β*. With a low power (<30) scale-free Topology Fit Index (TFI) of 0.9 or more, the topology of the gene coexpression network was considered scale-free and there were no batch effects. Thus, the power *β* = 8 was chosen [34]. After that, the Topological Overlap Measure (TOM) was adopted to detect network modules [35]. The minimum module size was fixed at 30, and the other parameters were fixed at their default values. At the same time, the first principal component of a given module was measured to calculate the module eigengene to represent each module. Different modules were indicated by different colors. The gray module was used to indicate the group of genes that was not categorized into any modules.

Subsequently, Module-Trait Relationships (MTRs) were applied to establish the vital relationship among module eigengenes and TNBC subtypes categorized in the GSE76124 database. Gene Significance (GS) was calculated to identify the relevance of traits and genes. Module Membership (MM) was evaluated to confirm the relevance of the expression profile and every module eigengene. At last, the genes with high GS and significant MM were identified in the TNBC subtype.

### 2.3. Differentially Expressed Gene Screening

The DEGs between the AR-positive subtype and the other 3 subtypes of TNBC tissues were identified by use of the R package limma. The DEGs were defined as the gene that met the cut-off criteria of |log2fold change (FC)| > 1 and *P* value < 0.05. Afterward, Venn diagrams were adopted to select the overlapped DEGs. Eventually, the final overlapping DEGs were selected from the intersection of the WGCNA-identified module genes (purple module genes) and aforementioned common DEGs for subsequent function analysis.

### 2.4. Gene Functional Annotation Analysis

The final DEGs were selected to conduct a functional enrichment analysis. The online database DAVID (https://david.ncifcrf.gov/) was employed to perform the GO and KEGG pathway enrichment analyses. Three categories were included in GO analysis, cellular component (CC), biological process (BP), and molecular functions (MF). Various pathway information of the genes were contained in KEGG analysis [36].

### 2.5. Protein-Protein Interaction (PPI) Analysis

The PPI network for final overlapping DEGs was established by the STRING database with a combined interaction score > 0.4 and visualized via Cytoscape software (version 3.8.2). Next, the cytoHubba was employed to select the hub genes according to the network. The first 10 genes selected with the MCC method were defined as core genes [37, 38].

### 2.6. Survival Analysis and Validation of the Hub Genes

The prognostic value of the identified hub genes in TNBC was assessed by the online database KM plotter that included the gene expression profile and corresponding prognostic information of patients from the TCGA and GEO databases. In the current study, the parameters were arranged as follows: (1) the negative expression of ER, PR, and HER-2; (2) only JetSet best probe set. Besides, the GEPIA platform was employed to confirm the mRNA expression levels of the core genes in tumor and normal breast tissues (|log2FC| cut − off > 1 and *P* value cut-off < 0.01) [39]. Another dataset from the GEO database, GSE167213, was used as the validation group. The expression of hub genes was calculated between AR-positive TNBC samples and other subtypes of TNBC samples.

### 2.7. Identification of Candidate miRNAs

The targeted miRNAs of TFF1 were predicted through online databases TargetScan and ENCORI. The parameters of TargetScan were default. The parameters of ENCORI were set as follows: (1) CLIP data: high stringency (≥3); (2) Degradome data: with or without data; (3) Pan-cancer: one cancer type. Accordingly, the intersection miRNAs of TargetScan and ENCORI were further selected as the candidate miRNAs of TFF1 [40].

### 2.8. Survival Analysis and Validation of the Candidate miRNAs

The relevance of TFF1 and its candidate miRNAs was confirmed by ENCORI. Meanwhile, the selected miRNA expression levels were compared among tumor and normal breast tissues. TFF1 was found to have significant overexpression in AR-positive TNBC, which was associated with the unfavorable prognosis. Hence, the miRNAs moderating TFF1 were hypothesized to be related to better prognosis of TNBC. Furthermore, the prognostic correlation of the selected miRNAs was evaluated by the KM plotter (the paraments were set as TCGA, TNBC, and OS).

### 2.9. The Interaction of Drug-Hub Gene

DGIdb (http://www.dgidb.org/search_interactions; version 3.0.2) was adopted to select the drugs on the basis of the core genes served as potential therapeutic targets. The interaction network of the hub genes and possible drugs was created by means of the Cytoscape software [41].

## 3. Results

### 3.1. Establishment of the Coexpression Module and Identification of the Core Module in TNBC

**Figure 1:**
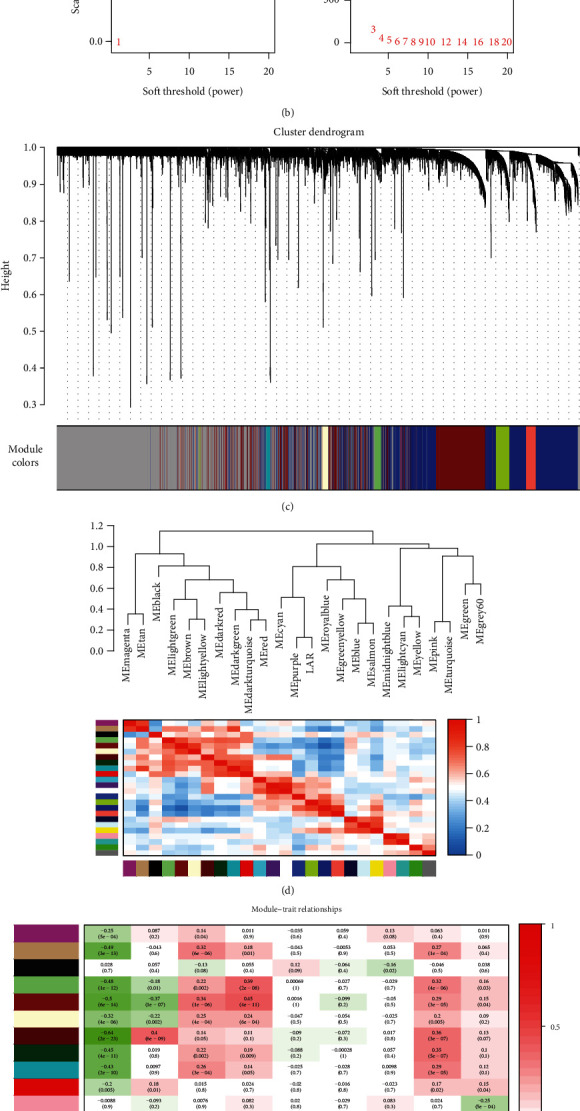
Identification of significant gene modules. (a) Clustering dendrograms of genes. Color intensity varies with MES, LAR, BLIS, BLIA, T, N, M, age, and BMI. (b) Scale-free fit index (left) and the mean connectivity (right) for soft-thresholding powers. When *β* was set at 8, the scale-free network was constructed in the GSE76124 database. (c) Clustering dendrograms of genes based on dissimilarity topological overlap and module colors in GSE76124 database. 24 coexpression modules were established and marked by different colors. (d) Visualizing the gene network using a heat map plot. The module eigengene dendrogram and heat map verified that the purple module was positively correlated with AR-positive TNBC. (e) Analysis of module-trait relationships of TNBC based on the dataset GSE76124. Pearson correlation coefficient matrix was calculated between traits and modules. The correlation coefficient of each module and the corresponding *P* value were displayed. A positive correlation between the purple module (containing 227genes) and the AR-positive TNBC was indicated with a *P* < 0.05 (correlation coefficient = 0.87, *P* < 0.01). (f) A scatter plot of GS for AR-positive TNBC and the MM in the purple module. Intramodular analysis indicated that the genes in the purple module had a high correlation with AR-positive TNBC, with *P* = 4.6*e* − 54 and correlation = 0.81.

A weighted coexpression network was built through R package WGCNA. The 198 samples of the GSE76124 database were clustered to filter outliers for follow-up study, and 2 outlier samples (GSM1974605 and GSM1974616) were removed by setting the height line at 150; then, the new cluster was proposed and a characteristic heat map was exhibited based on the subtypes of TNBC (Figure 1(a)). Subsequently, the power of *β* = 8 (scale-free R2 = 0.90) was selected as the soft-thresholding parameter to make sure of a scale-free network (Figure 1(b)). From 23,519 genes, 24 modules were distinguished and every one was represented with an individual color in the hierarchical clustering dendrogram (Figure 1(c)). The module-trait association was evaluated by the relevance between the module eigengene and the clinical characteristics including TNBC subtypes. Interestingly, the positive correlation between the purple module (containing 227 genes) and the AR-positive TNBC was indicated with the *P* < 0.05 (correlation coefficient = 0.87, *P* < 0.01) (Figure 1(e)). Subsequently, the module eigengene dendrogram and heat map indicated that the purple module was positively related to AR-positive TNBC (Figure 1(d)). After that, the scatterplot of GS vs. MM was drawn based on the coexpression purple module (Figure 1(f)). Consequently, the purple module was selected as the candidate module for further analysis.

### 3.2. The Analysis of DEGs

**Figure 2:**
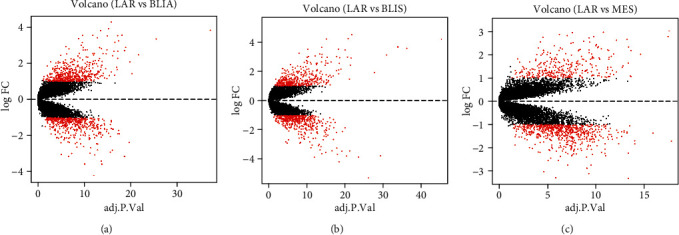
Volcano plot of gene expression in GSE76124 database. The upregulated and downregulated genes were represented as red. (a) LAR vs. BLIA, (b) LAR vs. BLIS, and (c) LAR vs. MES.

**Figure 3:**
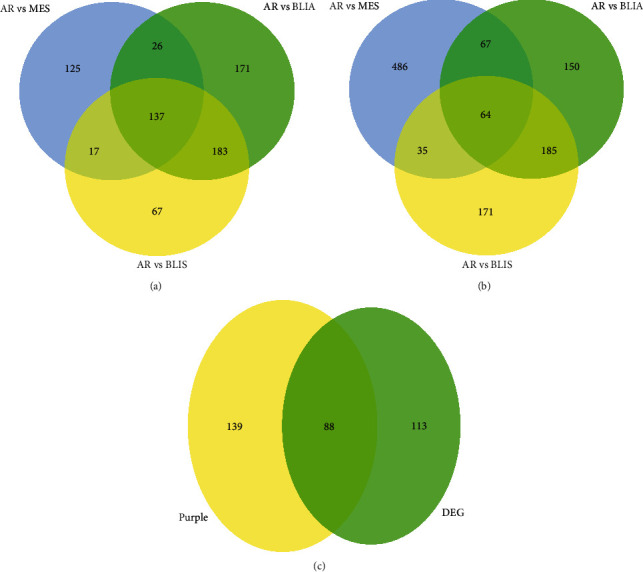
Venn diagram displaying the number of genes in different groups. (a) Compared with the other three TNBC subtype tissues, 137 common DEGs were upregulated (*P* < 0.05, log2FC > 1) in AR-positive TNBC tissues. (b) Compared with the other three TNBC subtype tissues, 64 common DEGs were downregulated (*P* < 0.05, log2FC < −1) in AR-positive TNBC tissues. (c) 88 overlapping genes were selected from the intersection of the purple module genes identified by WGCNA and common DEGs identified by limma.

On the basis of the clinical traits, 198 TNBC samples in the GSE76124 dataset were divided into 4 groups, LAR, MES, BLIA, and BLIS. The group LAR tissue dataset and the other three-group tissue datasets were analyzed to identify DEGs. Group LAR vs. Group MES, Group LAR vs. Group BLIA, and Group LAR vs. Group BLIS were analyzed, indicating 957 (652 downregulated and 305 upregulated), 983 (466 downregulated and 517 upregulated), and 859 (455 downregulated and 404 upregulated) DEGs (Figure 2). Afterwards, Venn diagrams were used to identify the overlapped DEGs. As a result, 201 overlapped DEGs were discovered, in which 64 were downregulated (*P* < 0.05, log2FC < −1) and 137 were upregulated (*P* < 0.05, log2FC > 1) (Figures 3(a) and 3(b)). Eventually, 88 DEGs were selected from the intersection of the purple module genes identified by WGCNA and aforementioned common DEGs (Figure 3(c)).

### 3.3. GO and KEGG Pathway Enrichment Analyses

**Figure 4:**
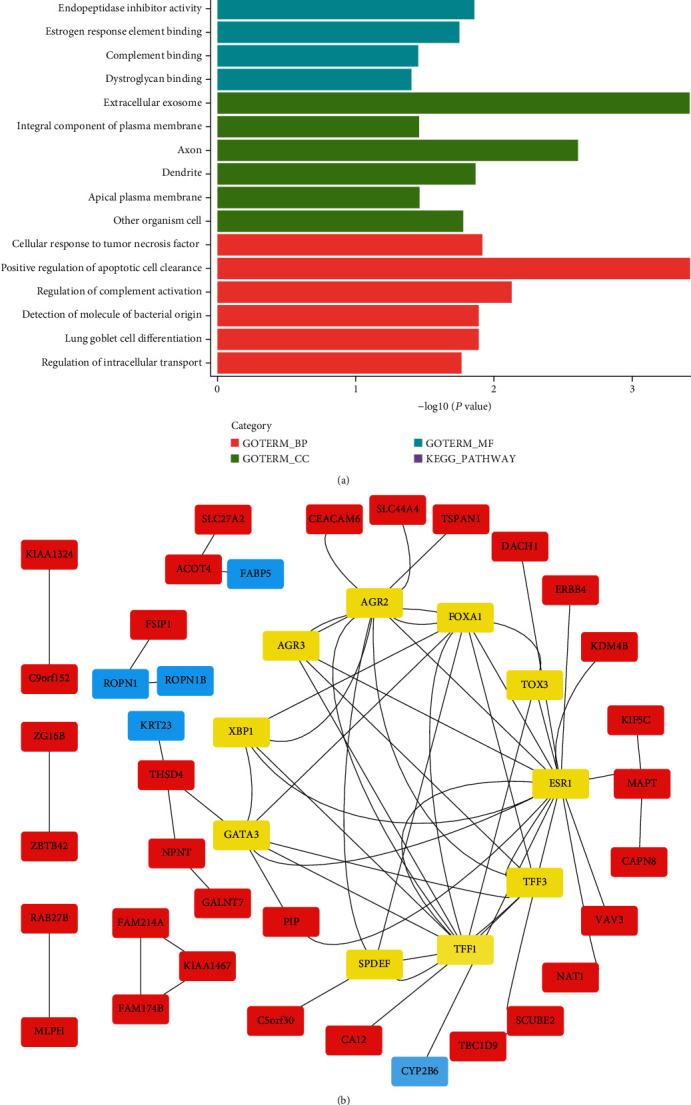
Gene functional annotation of the set of 88 shared genes and the PPI network of hub genes. (a) Kyoto Encyclopedia of Genes and Genomes (KEGG) pathway enrichment analysis of the 88 shared genes. *P* < 0.05. GO analysis of the 88 shared genes including biological process (BP), cellular component (CC), and molecular function (MF). (b) Protein-protein interaction network of hub gene based on the STRING database. The upregulated DEGs were represented as red and the downregulated DEGs were represented as blue. The top 10 genes were selected as candidate hub genes based on MCC algorithm, represented as yellow.

GO enrichment analysis was applied to the 88 selected DEGs to find the potential biological functions. As shown in Figure 4(a) (*P* value < 0.05), biological processes involved in DEGs are positive regulation of apoptotic cell clearance, cellular response to tumor necrosis factor, regulation of complement activation, detection of molecule of bacterial origin, regulation of intracellular transport, and lung goblet cell differentiation. Cellular components of DEGs are integral component of plasma membrane, extracellular exosome, axon, dendrite, apical plasma membrane, and other organism cells. Molecular functions involved in DEGs are carbohydrate binding, epidermal growth factor receptor binding, endopeptidase inhibitor activity, estrogen response element binding, complement binding, and dystroglycan binding. KEGG pathway enrichment analysis was applied to the 88 DEGs, indicating that DEGs was mostly enriched in the metabolic pathways.

### 3.4. Establishment of PPI Network and Identification of Hub Genes

The PPI network for the 88 overlapping DEGs was established by STRING and displayed in Cytoscape, containing 81 nodes and 63 edges. Subsequently, the topological analysis methods were employed to pick out hub genes, and the top 10 genes were identified by the MCC method. As a result, TFF1, FOXA1, ESR1, AGR2, TFF3, AGR3, GATA3, XBP1, SPDEF, and TOX3 were selected, and they were all upregulated (Figure 4(b)).

### 3.5. Expression and Survival Analysis for Hub Genes

**Figure 5:**
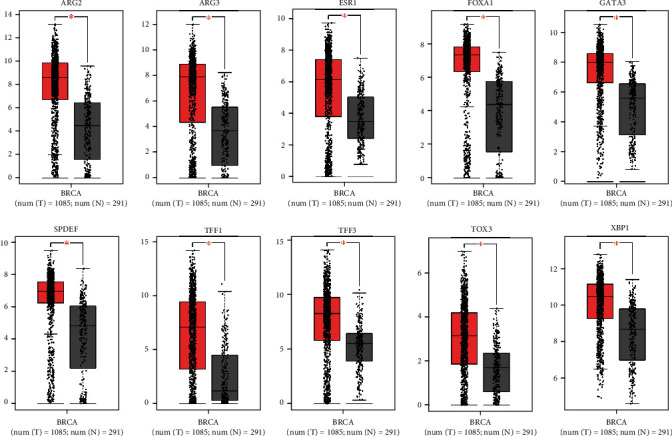
The expression levels of the 10 hub genes in tumor and normal tissues. The expression levels of the 10 hub genes were detected by GEPIA (|log2FC| cut − off value = 1 and *P* value cut-off value = 0.01). Contrasting to normal samples, all of the hub genes were highly expressed in breast cancer samples (*P* < 0.01). The tumor tissues were marked by red, and the normal tissues were marked by gray.

**Figure 6:**
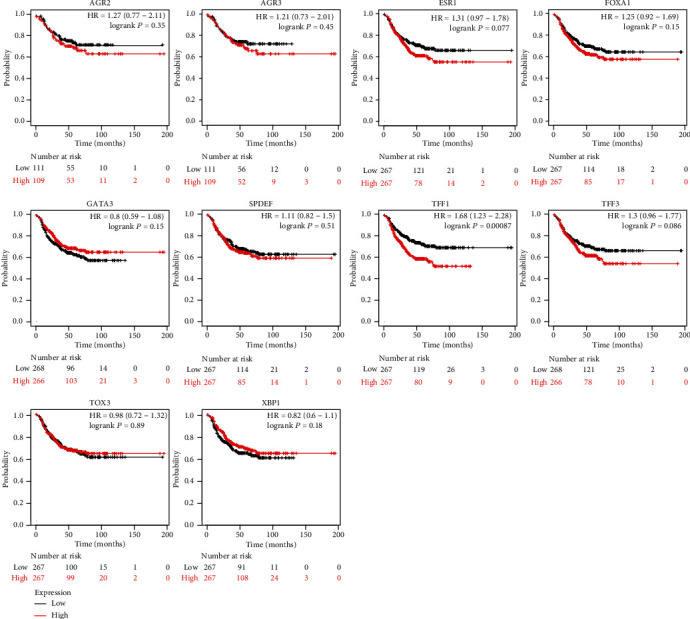
Associated candidate gene expression and overall survival time using the Kaplan–Meier plotter online platform. The Kaplan–Meier test *P* value < 0.05: TFF1. The Kaplan–Meier test *P* value > 0.05: FOXA1, ESR1, AGR2, TFF3, AGR3, GATA3, XBP1, SPDEF, and TOX3.

GEPIA was employed to detect the expression of 10 hub genes (the paraments were set as |log2FC| cut − off value = 1 and *P* value cut-off value = 0.01). The outcomes manifested that the 10 core gene expression levels were statistically higher in tumor tissues than in normal breast tissues (Figure 5). Besides, the survival analysis for the aforementioned 10 hub genes were performed via KM plotter, indicating that only TFF1 was related to significantly poorer survival outcome (*P* < 0.05) in TNBC (Figure 6). Therefore, TFF1 was marked as the key hub gene. In the validation group, the GSE167213 dataset, the expression of TFF1 was calculated between AR-positive and other subtypes of TNBC samples. As shown in Supplemental Figure 1, the expression level of TFF1 in AR-positive TNBC was significantly higher than that in other subtypes.

### 3.6. Recognition of Candidate miRNAs

**Figure 7:**
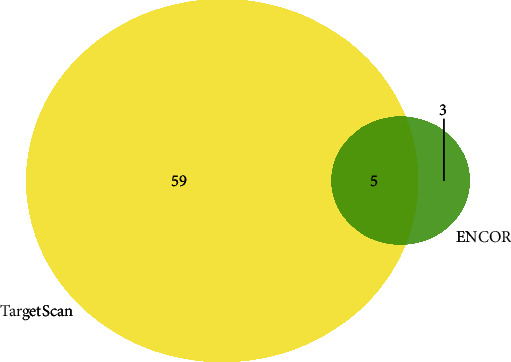
Validation of the overlapped miRNAs in the two databases (ENCORI and TargetScan) visualized by Venn diagram. A total of 8 miRNAs regulating TFF1 were predicted by ENCORI and 64 miRNAs were predicted by TargetScan. 5 common miRNAs regulating TFF1 were identified via the Venn diagrams.

TargetScan and ENCORI were applied to screen the targeted miRNAs of TFF1. 64 miRNAs regulating TFF1 were forecasted by TargetScan, and 8 were forecasted by ENCORI. Finally, the 5 overlapped miRNAs regulating TFF1 were selected through Venn diagrams (Figure 7). As revealed in Table 1, hsa-miR-187-3p, hsa-miR-520g-3p, hsa-miR-520h, and hsa-miR-2278 were selected as the candidate miRNAs of TFF1 (*P* < 0.05). Based on the *P* value > 0.05, hsa-miR-1295a was excluded.

### 3.7. Candidate miRNA Survival Analysis and Expression Analysis

**Figure 8:**
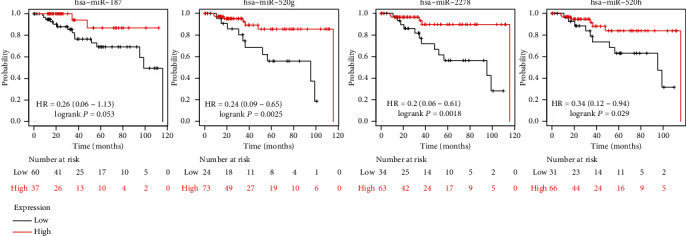
Associated miRNA expression and overall survival time using the K-M plotter online platform. hsa-miR-520g-3p, hsa-miR-520h, and hsa-miR-2278 were statistically associated with the poor prognosis of TNBC (*P* < 0.05), while hsa-miR-187-3p was excluded without statistical significance (*P* > 0.05).

**Figure 9:**
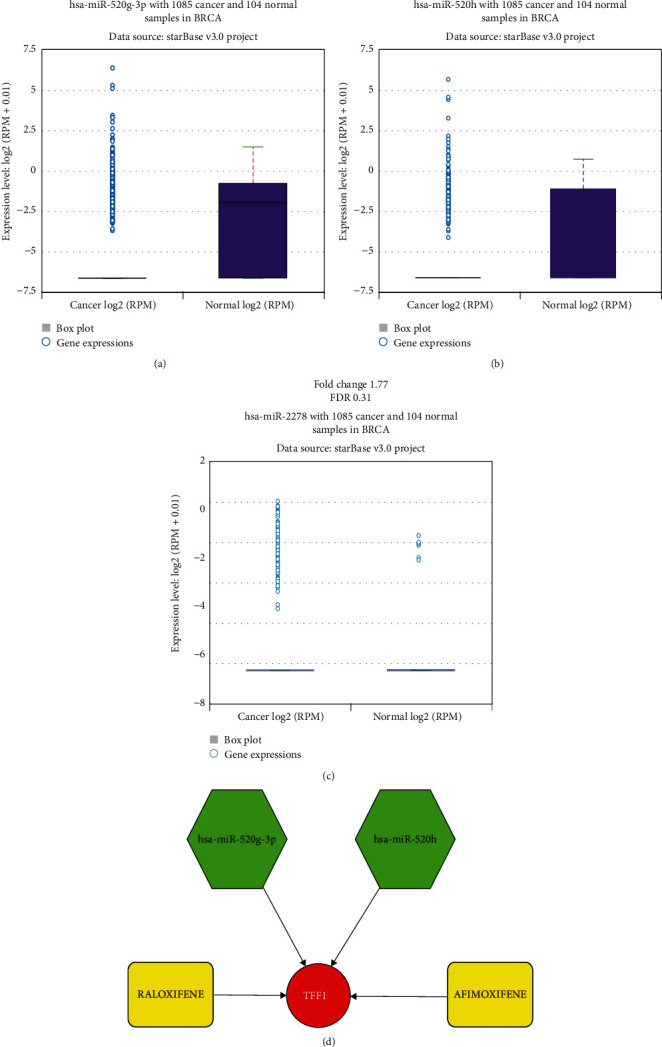
The expression levels of candidate miRNAs and the interaction network between the hub gene, targeted miRNAs, and drugs. (a) The expression levels of hsa-miR-520g-3p. (b) The expression levels of hsa-miR-520h. (c) The expression levels of hsa-miR-2278. There was no significant difference between the expressions of hsa-miR-2278 in breast cancer tissues and normal tissues. (d) The interaction of drug gene and miRNA-hub gene networks.

The KM plotter was applied to the 4 candidate miRNAs for survival analysis. hsa-miR-520g-3p, hsa-miR-520h, and hsa-miR-2278 were statistically related to poor prognostic outcomes of TNBC (*P* < 0.05). Although hsa-miR-187-3p was correlated with the poor prognosis, it was excluded without statistical significance (Figure 8). Eventually, ENCORI pan-cancer analysis was conducted to indicate the differences of hsa-miR-520g-3p, hsa-miR-520h, and hsa-miR-2278 expression between tumor and normal breast tissues. Figures 9(a)–9(c) illustrate that hsa-miR-520g-3p and hsa-miR-520h were significantly downregulated in breast cancer samples. There was no noticeable difference in the expression of has-miR-2278 in breast cancer and normal samples.

### 3.8. The Interaction of Drug-Gene Network

Two potential drugs for AR-positive TNBC patients were suggested by drug-gene interactions. In the current study, based on the significant outcomes of survival analysis, FTT1 was selected as the hub gene, meanwhile AFIMOXIFENE (4-4-hydroxytamoxifen) and raloxifene were identified as the potential targeted drugs. However, only raloxifene was approved by the FDA. The drug-gene network was visualized by Cytoscape (Figure 9(d)).

## 4. Discussion

As a malignant disease whose pathogenesis is not fully understood, breast cancer is highly heterogeneous in terms of patient prognosis and tumor genetics. TNBC is more aggressive than other subtypes of BC, and patients suffering from TNBC showed a higher mortality rate [11, 42]. The heterogeneous nature of TNBC makes the treatment of tumors more challenging. It is essential to understand the regulatory mechanisms behind the development of TNBC so as to enhance the therapeutic response of tumors. In some studies, a subgroup of TNBC had been established with AR expression, finding that AR was expressed in 15% to 35% of all TNBC, indicating that AR is able to be a possible target of TNBC treatment. Observations from these studies also revealed the vital role of AR in promoting the migration and invasion of TNBC cells. Actually, AR is able to perform multiple roles in BC progression and serve as an effective target for the management of AR-positive TNBC patients in the clinical setting [12, 21, 43–46].

As a new tool that is based on complex algorithms, WGCNA for network modelling enables the identification of multiple biological associations of biological networks with their phenotypes. Recently, WGCNA was employed in several studies of refractory diseases to further clarify the regulatory mechanisms, including Alzheimer's disease [47], familial combined hyperlipidemia [48], and BC [29]. In the current study, WGCNA methods and DEG analysis were applied to detect the differences between AR-positive TNBC and non-AR-positive TNBC samples, respectively.

The results of the WGCNA analysis identified critical modules of clinical significance and were screened for purple modules by conservation assessment. Subsequently, the overlapped genes of DEGs and the purple module were selected for further study.

After that, GO and KEGG analyses were applied to study the chiefly relevant biological pathway of the intersection genes, and a PPI network was created. GO analysis suggested that the intersection genes principally participated in such pathways, including positive regulation of apoptotic cell clearance, cellular response to tumor necrosis factor, regulation of complement activation, detection of molecule of bacterial origin, integral component of plasma membrane, extracellular exosome, carbohydrate binding, epidermal growth factor receptor binding, endopeptidase inhibitor activity, and estrogen response element binding. Several biological pathways have been confirmed in previous studies [49–51]. KEGG analysis suggested that metabolic pathways were markedly enriched. Interestingly, the study of TNBC conducted by Jia et al. also proposed vital enrichment pathways of cellular senescence [52].

Finally, TFF1, FOXA1, ESR1, AGR2, TFF3, AGR3, GATA3, XBP1, SPDEF, and TOX3 were selected as hub genes; these were all upregulated. The survival analysis for the aforementioned 10 genes was performed via KM plotter, indicating that only TFF1 was related to statistically poorer survival in TNBC. ENCORI and TargetScan were adopted to identify the candidate miRNA of TFF1. According to survival and expression analyses, hsa-miR-520g-3p and hsa-miR-520h were selected as the candidate miRNAs of TFF1 (*P* < 0.05).

Mammalian trefoil factors consisted of 3 stable secretory proteins, TFF1, TFF2 and TFF3, which were coexpressed together with mucins through the epithelial cells of the gastrointestinal tract [53]. TFF1 belongs to the trefoil factor family, that is, a classic secreted peptide released from gastric surface mucous cells. 60 amino acid residues made up human TFF1, including 7 cysteine residues [54]. However, TFF1, TFF2, and TFF3 were initially recognized as estrogen-responsive gene products in BC cells [55]. The study conducted by Yi et al. demonstrated that TFF1 expression was much lower in TNBC and positively correlated with breast cancer survival. Moreover, they found that serum concentrations of TFF1 were lower in TNBC sufferers compared to non-TNBC sufferers, which correlated with the clinical features of BC sufferers, for instance, ER, PR, and HER2 status [56], whereas another study of BC reported that TFF1 was positively related to Circ-TFF1, and both of them were upregulated. In vitro, knockdown of Circ-TFF1 blocked BC cell growth, invasion, migration, and EMT while in vivo limiting tumor proliferation [57]. In addition, TFF1 was also considered as a biomarker of metastatic colon carcinoma [58]. Moreover, some studies indicated that TFF1 played an important role in the interacting of H. pylori and epithelial cells and related to gastric cancer [54, 59]. Although several studies manifested that TFF1 was related to different kinds of carcinoma, few studies on TFF1 in AR-positive TNBC have been reported.

Afimoxifene (4-hydroxytamoxifen) and raloxifene were selected as the potential drugs, but only raloxifene was approved by FDA. Meanwhile, the mechanisms of these 2 drugs were still unknown in AR-positive TNBC. Afimoxifene (4-hydroxytamoxifen, tradename TamoGel) was a novel estrogen inhibitor being investigated for various estrogen-dependent conditions, such as gynecomastia and cyclic breast pain. A previous study about estrogen response element (ERE) indicated that across a wide range of 4-hydroxytamoxifen (OHT) concentrations, OHT-hER*α* was closely related to the pS2 ERE and weakly to the PI-9 ERU [60]. Raloxifene was an oral selective estrogen receptor modulator (SERM) with estrogenic effects on the bones and antiestrogenic effects on the uterus and mammary gland. The observations of some studies demonstrated that the risk of invasive BC was decreased among postmenopausal women with osteoporosis during treatment of raloxifene [61]. Meanwhile, raloxifene was reported to be associated with vascular relaxing properties and treatment of postmenopausal women with schizophrenia [62, 63]. Wu et al. found that tamoxifen was associated with the induction of autophagy in TNBC cells, which was related to the endoplasmic reticulum stress and AMPK/mTOR [64]. The TNBC mouse models were used by Taurin and colleagues to evaluate the therapeutic value of raloxifene, suggesting that raloxifene (0.85 mg/kg) prevented tumor proliferation and led to tumor regression. Moreover, raloxifene was reported to promote EGFR translocation into endosomes in vitro, thereby reducing cell migration, invasiveness, and tumorigenicity [65]. Besides, another study in SERMs indicated that tamoxifen inhibited cell migration and enhanced chemosensitivity of mesenchymal TNBC cells by reversing their EMT-like property [66]. Nevertheless, to our knowledge, there are few studies in AR-positive TNBC. Further experiment should be conducted to explore the mechanisms of the candidate drugs in AR-positive TNBC.

According to the features of hub genes in terms of expression, biological function, signaling pathway, and previous associated studies, we considered that TFF1, hsa-miR-520g-3p, and hsa-miR-520h were likely to play vital roles in AR-positive TNBC and could be considered as potential biomarkers. Nevertheless, several limitations of our work should be noticed. First of all, the shared sources of data from the GEO and TCGA databases were only analyzed through a series of bioinformatics methods and no in vivo or in vitro experiments were performed. Secondly, this research only initially revealed the expression levels of TFF1 and hsa-miR-520g-3p and hsa-miR-520h in AR-positive TNBC but rarely addressed the signaling pathways and functional mechanisms. We only revealed the modulation relationship between them without information of details and regulation mechanisms. Hence, more prospective research is needed to validate the value of TFF1, hsa-miR-520g-3p, and hsa-miR-520h in AR-positive TNBC, and their relationships should be further investigated by wet assays.

## 5. Conclusion

In summary, our study focused on AR-positive relevant genes in TNBC. The vital gene modules and candidate genes related to AR-positive TNBC were identified by WGCNA and other bioinformatics methods. This study suggested that hsa-miR-520g-3p, hsa-miR-520h, and TFF1 could have remarkably potential diagnostic and prognostic values in AR-positive TNBC. TFF1, hsa-miR-520g-3p, and hsa-miR-520h are able to be novel therapeutic targets. Our findings offer further insights into the therapy of AR-positive TNBC. In the future, deeper molecular mechanism studies of novel core genes in AR-positive TNBC are required, and associated experimental models based on core genes should be established for early detection, risk estimation, prognosis determination, and targeted treatment of AR-positive TNBC.

## Figures and Tables

**Table 1:** Correlation between miRNA-TFF1 pairs identified by ENCORI database. hsa-miR-187-3p, hsa-miR-520g-3p, hsa-miR-520h, and hsa-miR-2278 were selected as the candidate miRNAs of TFF1 (*P* < 0.05).

No.	miRNA	Coefficient-*R*	*P* value
1	Hsa-miR-187-3p	-0.167	3.28*E* − 08
2	Hsa-miR-520g-3p	-0.11	2.73*E* − 04
3	Hsa-miR-520h	-0.094	1.85*E* − 03
4	Hsa-miR-1295a	-0.05	1.01*E* − 01
5	Hsa-miR-2278	-0.06	4.87*E* − 02

## Data Availability

The RNA-seq data and corresponding clinical information used to support the findings of this study are available on the GEO database (https://www.ncbi.nlm.nih.gov/geo/).

## Supplementary Materials

Supplementary 1 Supplemental Figure 1: the expression of TFF1 in the TNBC samples from the GSE167213 dataset. The expression levels of TFF1 in AR-positive TNBC were higher than those in the other subtypes of TNBC (*P* < 0.05).

Supplementary 2 Supplemental table 1: the features of 198 TNBC samples.

## References

[1] Siegel R., Miller K., Jemal A. (2020). Cancer statistics, 2020. *CA: a Cancer Journal for Clinicians*.

[2] Harbeck N., Gnant M. (2017). Breast cancer. *Lancet*.

[3] Zhao M., Scott S., Evans K. (2021). Combining neratinib with CDK4/6, mTOR, and MEK inhibitors in models of HER2-positive cancer. *Clinical Cancer Research*.

[4] Kyndi M., Sørensen F., Knudsen H. (2008). Estrogen receptor, progesterone receptor, HER-2, and response to postmastectomy radiotherapy in high-risk breast cancer: the Danish Breast Cancer Cooperative Group. *Journal of Clinical Oncology*.

[5] Carey L., Winer E., Viale G., Cameron D., Gianni L. (2010). Triple-negative breast cancer: disease entity or title of convenience?. *Nature Reviews. Clinical Oncology*.

[6] Dietze E., Chavez T., Seewaldt V. (2018). Obesity and triple-negative breast cancer: disparities, controversies, and biology. *The American Journal of Pathology*.

[7] He Z., Xu Q., Wang X. (2018). RPLP1 promotes tumor metastasis and is associated with a poor prognosis in triple-negative breast cancer patients. *Cancer Cell International*.

[8] Lavoro A., Scalisi A., Candido S. (2022). Identification of the most common BRCA alterations through analysis of germline mutation databases: is droplet digital PCR an additional strategy for the assessment of such alterations in breast and ovarian cancer families?. *International Journal of Oncology*.

[9] Shoukry M., Broccard S., Kaplan J., Gabriel E. (2021). The emerging role of circulating tumor DNA in the management of breast cancer. *Cancers*.

[10] Hong H., Chuang C., Huang W. (2020). A panel of eight microRNAs is a good predictive parameter for triple-negative breast cancer relapse. *Theranostics*.

[11] Xie W., du Z., Chen Y. (2020). Identification of metastasis-associated genes in triple-negative breast cancer using weighted gene co-expression network analysis. *Evolutionary Bioinformatics Online*.

[12] Lehmann B., Bauer J., Chen X. (2011). Identification of human triple-negative breast cancer subtypes and preclinical models for selection of targeted therapies. *The Journal of Clinical Investigation*.

[13] Burstein M., Tsimelzon A., Poage G. (2015). Comprehensive genomic analysis identifies novel subtypes and targets of triple-negative breast cancer. *Clinical Cancer Research*.

[14] Gucalp A., Traina T. (2010). Triple-negative breast cancer. *Cancer Journal*.

[15] Chang C., Lee S., Yeh S., Chang T. (2014). Androgen receptor (AR) differential roles in hormone-related tumors including prostate, bladder, kidney, lung, breast and liver. *Oncogene*.

[16] Hickey T., Robinson J., Carroll J., Tilley W. (2012). Minireview: the androgen receptor in breast tissues: growth inhibitor, tumor suppressor, oncogene?. *Molecular Endocrinology*.

[17] Nicolás Díaz-Chico B., Germán Rodríguez F., González A. (2007). Androgens and androgen receptors in breast cancer. *The Journal of Steroid Biochemistry and Molecular Biology*.

[18] Sutton L., Cao D., Sarode V. (2012). Decreased androgen receptor expression is associated with distant metastases in patients with androgen receptor-expressing triple-negative breast carcinoma. *American Journal of Clinical Pathology*.

[19] McNamara K., Yoda T., Takagi K., Miki Y., Suzuki T., Sasano H. (2013). Androgen receptor in triple negative breast cancer. *The Journal of Steroid Biochemistry and Molecular Biology*.

[20] Agoff S., Swanson P., Linden H., Hawes S., Lawton T. (2003). Androgen receptor expression in estrogen receptor-negative breast cancer. *American Journal of Clinical Pathology*.

[21] Michmerhuizen A., Chandler B., Olsen E. (2020). Seviteronel, a novel CYP17 lyase inhibitor and androgen receptor antagonist, radiosensitizes AR-positive triple negative breast cancer cells. *Frontiers in Endocrinology*.

[22] Gasparini P., Fassan M., Cascione L. (2014). Androgen receptor status is a prognostic marker in non-basal triple negative breast cancers and determines novel therapeutic options. *PLoS One*.

[23] Giovannelli P., Di Donato M., Auricchio F., Castoria G., Migliaccio A. (2019). Androgens induce invasiveness of triple negative breast cancer cells through AR/Src/PI3-K complex assembly. *Scientific Reports*.

[24] Candido S., Tomasello B., Lavoro A., Falzone L., Gattuso G., Libra M. (2021). Novel Insights into Epigenetic Regulation of IL6 Pathway: In Silico Perspective on Inflammation and Cancer Relationship.

[25] Giambò F., Leone G., Gattuso G. (2021). Genetic and epigenetic alterations induced by pesticide exposure: integrated analysis of gene expression, microRNA expression, and DNA methylation datasets. *International Journal of Environmental Research and Public Health*.

[26] Zhang B., Horvath S. (2005). A general framework for weighted gene co-expression network analysis. *Statistical Applications in Genetics and Molecular Biology*.

[27] Jia R., Zhao H., Jia M. (2020). Identification of co-expression modules and potential biomarkers of breast cancer by WGCNA. *Gene*.

[28] Guo X., Xiao H., Guo S., Dong L., Chen J. (2017). Identification of breast cancer mechanism based on weighted gene coexpression network analysis. *Cancer Gene Therapy*.

[29] Zhang J., Wang L., Xu X. (2020). Transcriptome-based network analysis unveils eight immune-related genes as molecular signatures in the immunomodulatory subtype of triple-negative breast cancer. *Frontiers in Oncology*.

[30] Niu J., Huang Y., Liu X. (2020). Single-cell RNA-seq reveals different subsets of non-specific cytotoxic cells in teleost. *Genomics*.

[31] Zhu N., Hou J., Ma G., Guo S., Zhao C., Chen B. (2020). Co-expression network analysis identifies a gene signature as a predictive biomarker for energy metabolism in osteosarcoma. *Cancer Cell International*.

[32] Heber S., Sick B. (2006). Quality Assessment of Affymetrix GeneChip Data. *OMICS*.

[33] Langfelder P., Horvath S. (2008). WGCNA: an R package for weighted correlation network analysis. *BMC Bioinformatics*.

[34] DiLeo M., Strahan G., den Bakker M., Hoekenga O. (2011). Weighted correlation network analysis (WGCNA) applied to the tomato fruit metabolome. *PLoS One*.

[35] Li A., Horvath S. (2009). Network module detection: affinity search technique with the multi-node topological overlap measure. *BMC Research Notes*.

[36] Huang D. W., Sherman B., Lempicki R. (2009). Systematic and integrative analysis of large gene lists using DAVID bioinformatics resources. *Nature Protocols*.

[37] Szklarczyk D., Morris J., Cook H. (2017). The STRING database in 2017: quality-controlled protein-protein association networks, made broadly accessible. *Nucleic Acids Research*.

[38] Ma H., He Z., Chen J., Zhang X., Song P. (2021). Identifying of biomarkers associated with gastric cancer based on 11 topological analysis methods of CytoHubba. *Scientific Reports*.

[39] Tang Z., Li C., Kang B., Gao G., Li C., Zhang Z. (2017). GEPIA: a web server for cancer and normal gene expression profiling and interactive analyses. *Nucleic Acids Research*.

[40] Peterson S., Thompson J., Ufkin M., Sathyanarayana P., Liaw L., Congdon C. B. (2014). Common features of microRNA target prediction tools. *Frontiers in Genetics*.

[41] Yang D., He Y., Wu B. (2020). Integrated bioinformatics analysis for the screening of hub genes and therapeutic drugs in ovarian cancer. *Journal of Ovarian Research*.

[42] Li X., Yang J., Peng L. (2017). Triple-negative breast cancer has worse overall survival and cause-specific survival than non-triple-negative breast cancer. *Breast Cancer Research and Treatment*.

[43] Proverbs-Singh T., Feldman J., Morris M., Autio K., Traina T. (2015). Targeting the androgen receptor in prostate and breast cancer: several new agents in development. *Endocrine-Related Cancer*.

[44] Ni M., Chen Y., Lim E. (2011). Targeting androgen receptor in estrogen receptor-negative breast cancer. *Cancer Cell*.

[45] Cochrane D., Bernales S., Jacobsen B. (2014). Role of the androgen receptor in breast cancer and preclinical analysis of enzalutamide. *Breast Cancer Research*.

[46] Barton V., D'Amato N., Gordon M. (2015). Multiple molecular subtypes of triple-negative breast cancer critically rely on androgen receptor and respond to enzalutamide in vivo. *Molecular Cancer Therapeutics*.

[47] Miller J., Oldham M., Geschwind D. (2008). A systems level analysis of transcriptional changes in Alzheimer's disease and normal aging. *The Journal of neuroscience : the official journal of the Society for Neuroscience*.

[48] Plaisier C., Horvath S., Huertas-Vazquez A. (2009). A systems genetics approach implicates USF1, FADS3, and other causal candidate genes for familial combined hyperlipidemia. *PLoS Genetics*.

[49] Kruer T., Cummins T., Powell D., Wittliff J. (2013). Characterization of estrogen response element binding proteins as biomarkers of breast cancer behavior. *Clinical Biochemistry*.

[50] Fichter C., Przypadlo C., Buck A. (2017). A new model system identifies epidermal growth factor receptor–human epidermal growth factor receptor 2 (HER2) and HER2–human epidermal growth factor receptor 3 heterodimers as potent inducers of oesophageal epithelial cell invasion. *The Journal of Pathology*.

[51] Cao R., Li X., Liu Z. (2006). Integration of a two-phase partition method into proteomics research on rat liver plasma membrane proteins. *Journal of Proteome Research*.

[52] Jia D., Lu M., Jung K. (2019). Elucidating cancer metabolic plasticity by coupling gene regulation with metabolic pathways. *Proceedings of the National Academy of Sciences of the United States of America*.

[53] Wright N., Poulsom R., Stamp G. (1993). Trefoil peptide gene expression in gastrointestinal epithelial cells in inflammatory bowel disease. *Gastroenterology*.

[54] Znalesniak E., Salm F., Hoffmann W. (2020). Molecular alterations in the stomach of Tff1-Deficient mice: early steps in antral carcinogenesis. *International Journal of Molecular Sciences*.

[55] Masiakowski P., Breathnach R., Bloch J., Gannon F., Krust A., Chambon P. (1982). Cloning of cDNA sequences of hormone-regulated genes from the MCF-7 human breast cancer cell line. *Nucleic Acids Research*.

[56] Yi J., Ren L., Li D. (2020). Trefoil factor 1 (TFF1) is a potential prognostic biomarker with functional significance in breast cancers. *Biomedicine & pharmacotherapy = Biomedecine & pharmacotherapie*.

[57] Pan G., Mao A., Liu J., Lu J., Ding J., Liu W. (2020). Circular RNA hsa_circ_0061825 (circ-TFF1) contributes to breast cancer progression through targeting miR-326/TFF1 signalling. *Cell Proliferation*.

[58] Vocka M., Langer D., Petrtyl J. (2015). Trefoil factor family (TFF) proteins as potential serum biomarkers in patients with metastatic colorectal cancer. *Neoplasma*.

[59] Clyne M., May F. (2019). The interaction of Helicobacter pylori with TFF1 and its role in mediating the tropism of the bacteria within the stomach. *International Journal of Molecular Sciences*.

[60] Krieg A., Krieg S., Ahn B., Shapiro D. (2004). Interplay between estrogen response element sequence and ligands controls in vivo binding of estrogen receptor to regulated genes. *The Journal of Biological Chemistry*.

[61] Cummings S., Eckert S., Krueger K. (1999). The effect of raloxifene on risk of breast cancer in postmenopausal women: results from the MORE randomized trial. Multiple Outcomes of Raloxifene Evaluation. *JAMA*.

[62] Figtree G., Lu Y., Webb C., Collins P. (1999). Raloxifene acutely relaxes rabbit coronary arteries in vitro by an estrogen receptor-dependent and nitric oxide-dependent mechanism. *Circulation*.

[63] Labad J., Martorell L., Huerta-Ramos E. (2016). Pharmacogenetic study of the effects of raloxifene on negative symptoms of postmenopausal women with schizophrenia: a double-blind, randomized, placebo-controlled trial. *European Neuropsychopharmacology*.

[64] Wu S., Sun G., Cha T. (2016). CSC-3436 switched tamoxifen-induced autophagy to apoptosis through the inhibition of AMPK/mTOR pathway. *Journal of Biomedical Science*.

[65] Taurin S., Allen K., Scandlyn M., Rosengren R. (2013). Raloxifene reduces triple-negative breast cancer tumor growth and decreases EGFR expression. *International Journal of Oncology*.

[66] Wang Q., Cheng Y., Wang Y. (2017). Tamoxifen reverses epithelial-mesenchymal transition by demethylating miR-200c in triple-negative breast cancer cells. *BMC Cancer*.

