# Supplementary material for: Integrated Bioinformatics Analysis for the Screening of Hub Genes and Therapeutic Drugs in Androgen Receptor-Positive TNBC
Source: Dis Markers. 2022 Sep 14;2022:4964793. doi: 10.1155/2022/4964793 (PMC9493148; doi:10.1155/2022/4964793)
Supplement: Supplementary 1 — Supplemental Figure 1: the expression of TFF1 in the TNBC samples from the GSE167213 dataset. The expression levels of TFF1 in AR-positive TNBC were higher than those in the other subtypes of TNBC (P < 0.05). [file 4964793.f1.pdf]

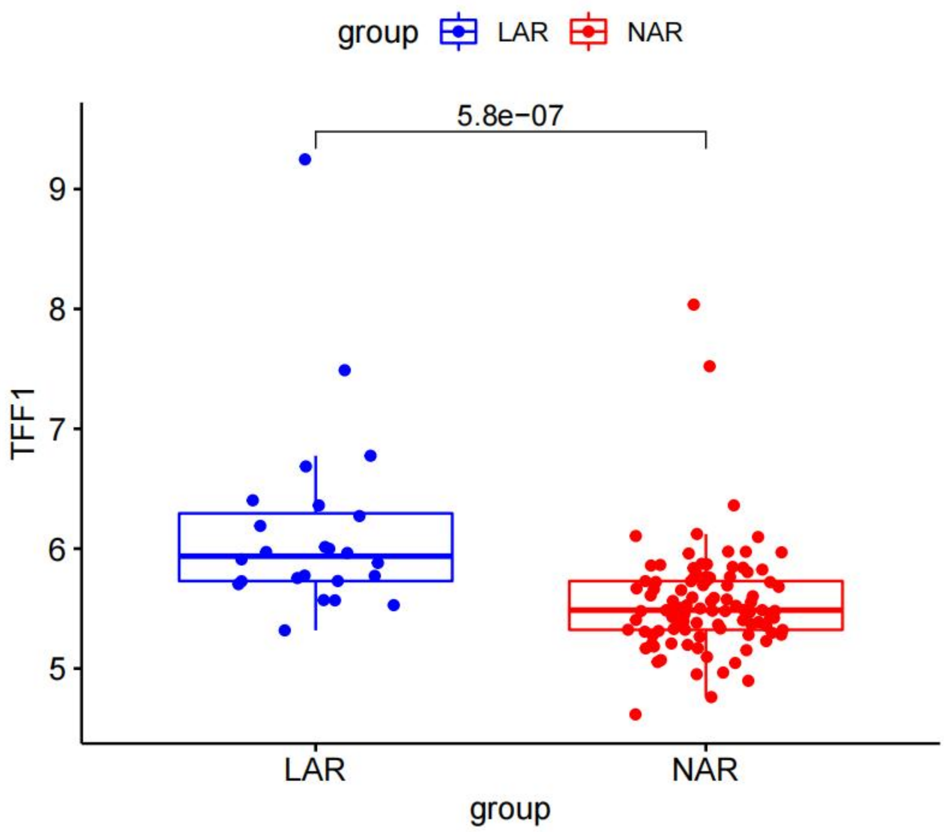

Supplemental Fig 1. The expression of TFF1 in the TNBC samples from GSE167213 dataset. The expression levels of TFF1 in AR-positive TNBC were higher than in the other subtypes of TNBC ($P<0.05$).
