# Supplementary material for: Integrated Bioinformatics Analysis for the Screening of Hub Genes and Therapeutic Drugs in Androgen Receptor-Positive TNBC
Source: Dis Markers. 2022 Sep 14;2022:4964793. doi: 10.1155/2022/4964793 (PMC9493148; doi:10.1155/2022/4964793)
Supplement: Supplementary 2 — Supplemental table 1: the features of 198 TNBC samples. [file 4964793.f2.pdf]

The features of 198 TNBC samples

sample	Title	group	ajcc stage (7th edition, 2010)	age (years)	body mass index	menopausal status
GSM1974763	S3-H99C	Luminal-AR (LAR)	T2NXM0	50	33	Post-Menopausal
GSM1974762	S3-H98C	Mesenchymal (MES)	T2N1M0	71	20	Post-Menopausal
GSM1974761	S3-H97C	Basal-Like Immune-Suppressed (BLIS)	T3N1M0	42	46	Pre-Menopausal
GSM1974760	S3-H96C	Basal-Like Immune-Suppressed (BLIS)	T2N0M0	39	27	Pre-Menopausal
GSM1974759	S3-H95C	Basal-Like Immune-Suppressed (BLIS)	T2N1M0	51	27	Post-Menopausal
GSM1974758	S3-H94C	Basal-Like Immune-Activated (BLIA)	T4bN2MX	47	NA	NA
GSM1974757	S3-H93C	Mesenchymal (MES)	T2NXM0	39	21	Pre-Menopausal
GSM1974756	S3-H230	Mesenchymal (MES)	T1NXMX	66	22	Post-Menopausal
GSM1974755	S3-H229	Basal-Like Immune-Activated (BLIA)	T2N0M0	72	29	Post-Menopausal
GSM1974754	S3-H228	Basal-Like Immune-Activated (BLIA)	T2NXM0	49	25	Pre-Menopausal
GSM1974753	S3-H227	Luminal-AR (LAR)	T1cN0M0	48	26	NA
GSM1974752	S3-H226	Mesenchymal (MES)	T2NXMX	57	28	Post-Menopausal
GSM1974751	S3-H225	Luminal-AR (LAR)	T2N2aM0	45	19	NA
GSM1974750	S3-H224	Basal-Like Immune-Suppressed (BLIS)	T2N1M0	49	24	Pre-Menopausal
GSM1974749	S3-H222	Basal-Like Immune-Activated (BLIA)	T1cNXM0	85	21	Post-Menopausal
GSM1974748	S3-H221	Mesenchymal (MES)	T2NXM0	48	22	Pre-Menopausal
GSM1974747	S3-H220	Basal-Like Immune-Activated (BLIA)	T2NXM0	53	32	Menopausal
GSM1974746	S3-H219	Basal-Like Immune-Suppressed (BLIS)	T2N0M0	39	20	Post-Menopausal
GSM1974745	S3-H218	Basal-Like Immune-Suppressed (BLIS)	T2N0M0	73	25	NA
GSM1974744	S3-H217	Basal-Like Immune-Activated (BLIA)	T2NXM0	51	30	Menopausal
GSM1974743	S3-H216	Mesenchymal (MES)	T2N0M0	58	25	Post-Menopausal
GSM1974742	S3-H215	Basal-Like Immune-Suppressed (BLIS)	T2N1M0	73	36	Post-Menopausal
GSM1974741	S3-H214	Luminal-AR (LAR)	T2N0M0	64	29	Post-Menopausal
GSM1974740	S3-H213	Basal-Like Immune-Suppressed (BLIS)	T2N0M0	46	36	Pre-Menopausal
GSM1974739	S3-H212	Luminal-AR (LAR)	T2NXM0	53	26	Menopausal
GSM1974738	S3-H211	Basal-Like Immune-Activated (BLIA)	T2NXMX	59	27	Post-Menopausal
GSM1974737	S3-H210	Basal-Like Immune-Suppressed (BLIS)	T1cN1M0	51	29	NA
GSM1974736	S3-H209	Mesenchymal (MES)	T1NXM0	57	32	Post-Menopausal
GSM1974735	S3-H208	Luminal-AR (LAR)	T1cNXM0	58	29	Post-Menopausal
GSM1974734	S3-H207	Mesenchymal (MES)	T2NXM0	68	32	Post-Menopausal
GSM1974733	S3-H206	Mesenchymal (MES)	T2NXMX	47	26	Pre-Menopausal
GSM1974732	S3-H205	Basal-Like Immune-Activated (BLIA)	T2NXM0	48	24	Pre-Menopausal
GSM1974731	S3-H204	Basal-Like Immune-Suppressed (BLIS)	T2N0M0	54	32	Post-Menopausal
GSM1974730	S3-H203	Luminal-AR (LAR)	T2NXM0	80	20	Post-Menopausal
GSM1974729	S3-H202	Luminal-AR (LAR)	T2N0MX	48	NA	Pre-Menopausal
GSM1974728	S3-H201	Luminal-AR (LAR)	T2N0M0	58	27	Post-Menopausal
GSM1974727	S3-H200	Basal-Like Immune-Suppressed (BLIS)	T2NXM0	46	27	Pre-Menopausal
GSM1974726	S3-H199	Basal-Like Immune-Suppressed (BLIS)	T2NXM0	65	28	Post-Menopausal
GSM1974725	S3-H198	Mesenchymal (MES)	T2N1MX	59	44	Post-Menopausal

GSM1974724	S3-H197	Basal-Like Immune-Activated (BLIA)	T2NXM0	48	25	Pre-Menopausal
GSM1974723	S3-H196	Mesenchymal (MES)	T2N1M0	64	22	Menopausal
GSM1974722	S3-H194	Basal-Like Immune-Suppressed (BLIS)	T2N0M0	80	21	Post-Menopausal
GSM1974721	S3-H193	Basal-Like Immune-Activated (BLIA)	T2N1M0	66	NA	Post-Menopausal
GSM1974720	S3-H191	Luminal-AR (LAR)	T2NXMX	35	24	Pre-Menopausal
GSM1974719	S3-H190	Mesenchymal (MES)	T2NXMX	67	30	Post-Menopausal
GSM1974718	S3-H189	Basal-Like Immune-Activated (BLIA)	T2N1M0	87	NA	Post-Menopausal
GSM1974717	S3-H188	Mesenchymal (MES)	T2NXMX	68	32	Post-Menopausal
GSM1974716	S3-H187	Luminal-AR (LAR)	T4bN3M0	55	30	Post-Menopausal
GSM1974715	S3-H186	Basal-Like Immune-Suppressed (BLIS)	T1cN0M0	67	29	Post-Menopausal
GSM1974714	S3-H185	Mesenchymal (MES)	T2NXMX	44	24	Menopausal
GSM1974713	S3-H184	Basal-Like Immune-Suppressed (BLIS)	T3N1M0	43	30	Pre-Menopausal
GSM1974712	S3-H183	Basal-Like Immune-Activated (BLIA)	T1cN1M0	65	39	Post-Menopausal
GSM1974711	S3-H182	Basal-Like Immune-Suppressed (BLIS)	T2N0M0	44	22	Post-Menopausal
GSM1974710	S3-H181	Basal-Like Immune-Activated (BLIA)	T2N0M0	60	37	Post-Menopausal
GSM1974709	S3-H180	Basal-Like Immune-Activated (BLIA)	T1cN0MX	47	26	Pre-Menopausal
GSM1974708	S3-H179	Basal-Like Immune-Activated (BLIA)	T2N0M0	44	25	NA
GSM1974707	S3-H178	Basal-Like Immune-Suppressed (BLIS)	T2N0M0	39	25	Pre-Menopausal
GSM1974706	S3-H177	Luminal-AR (LAR)	T2N3M0	60	26	Post-Menopausal
GSM1974705	S3-H176	Basal-Like Immune-Suppressed (BLIS)	T2N0M0	53	21	Post-Menopausal
GSM1974704	S3-H175	Basal-Like Immune-Activated (BLIA)	T1cN1M0	36	28	Pre-Menopausal
GSM1974703	S3-H174	Mesenchymal (MES)	T2N0M0	50	NA	NA
GSM1974702	S3-H173	Basal-Like Immune-Suppressed (BLIS)	T2NXMX	36	17	Pre-Menopausal
GSM1974701	S3-H172	Basal-Like Immune-Activated (BLIA)	T2N0M0	38	22	Pre-Menopausal
GSM1974700	S3-H171	Basal-Like Immune-Activated (BLIA)	T2N0MX	70	NA	Post-Menopausal
GSM1974699	S3-H170	Basal-Like Immune-Activated (BLIA)	T1cN0M0	51	24	Post-Menopausal
GSM1974698	S3-H168	Basal-Like Immune-Suppressed (BLIS)	T2N1MX	48	20	Pre-Menopausal
GSM1974697	S3-H167	Basal-Like Immune-Activated (BLIA)	T2N1aM0	46	21	Pre-Menopausal
GSM1974696	S3-H166	Basal-Like Immune-Activated (BLIA)	T4bN0MX	60	NA	Post-Menopausal
GSM1974695	S3-H165	Basal-Like Immune-Suppressed (BLIS)	T4bN1M0	62	NA	Post-Menopausal
GSM1974694	S3-H164	Mesenchymal (MES)	T1N0M0	83	22	Post-Menopausal
GSM1974693	S3-H163	Basal-Like Immune-Suppressed (BLIS)	T2N1M0	32	NA	Pre-Menopausal
GSM1974692	S3-H162	Basal-Like Immune-Activated (BLIA)	T2N1MX	45	NA	Pre-Menopausal
GSM1974691	S3-H161	Basal-Like Immune-Activated (BLIA)	T3N2M0	61	24	NA
GSM1974690	S3-H160	Basal-Like Immune-Suppressed (BLIS)	T2N2M0	45	NA	Pre-Menopausal
GSM1974689	S3-H159	Luminal-AR (LAR)	T2NXMX	61	28	Post-Menopausal
GSM1974688	S3-H158	Luminal-AR (LAR)	T2N0M0	39	NA	NA
GSM1974687	S3-H157	Basal-Like Immune-Suppressed (BLIS)	T2N0MX	44	NA	NA
GSM1974686	S3-H156	Basal-Like Immune-Activated (BLIA)	T3N2M0	76	22	Post-Menopausal
GSM1974685	S3-H155	Luminal-AR (LAR)	T2N1M0	66	29	Post-Menopausal
GSM1974684	S3-H154	Mesenchymal (MES)	T2NXM0	49	22	Pre-Menopausal
GSM1974683	S3-H153	Basal-Like Immune-Suppressed (BLIS)	T2N1M0	57	24	Post-Menopausal
GSM1974682	S3-H152	Basal-Like Immune-Suppressed (BLIS)	T3N1MX	47	24	Post-Menopausal
GSM1974681	S3-H151	Luminal-AR (LAR)	T1cN0MX	41	NA	Pre-Menopausal

GSM1974680	S3-H150	Basal-Like Immune-Suppressed (BLIS)	T3N0MX	60	26	Post-Menopausal
GSM1974679	S3-H149	Basal-Like Immune-Activated (BLIA)	T1cN2M0	49	27	Menopausal
GSM1974678	S3-H148	Basal-Like Immune-Suppressed (BLIS)	T3N3M0	69	NA	NA
GSM1974677	S3-H147	Luminal-AR (LAR)	T1cN0MX	32	NA	Pre-Menopausal
GSM1974676	S3-H146	Luminal-AR (LAR)	T2N3MX	48	NA	Pre-Menopausal
GSM1974675	S3-H145	Basal-Like Immune-Activated (BLIA)	T1cNXM0	44	26	Pre-Menopausal
GSM1974674	S3-H144	Luminal-AR (LAR)	T1N0M0	50	33	Menopausal
GSM1974673	S3-H143	Luminal-AR (LAR)	T1N0M0	61	27	Post-Menopausal
GSM1974672	S3-H142	Mesenchymal (MES)	T2N1MX	54	27	Menopausal
GSM1974671	S3-H141	Basal-Like Immune-Activated (BLIA)	T2NXM0	47	34	Menopausal
GSM1974670	S3-H140	Basal-Like Immune-Activated (BLIA)	T3NXM0	66	38	Post-Menopausal
GSM1974669	S3-H139	Basal-Like Immune-Suppressed (BLIS)	T1cN0M0	66	26	Post-Menopausal
GSM1974668	S3-H138	Basal-Like Immune-Suppressed (BLIS)	T2N0M1	86	25	Post-Menopausal
GSM1974667	S3-H137	Basal-Like Immune-Suppressed (BLIS)	T2N2MX	50	NA	NA
GSM1974666	S3-H136	Mesenchymal (MES)	T2N1MX	69	32	Post-Menopausal
GSM1974665	S3-H135	Basal-Like Immune-Suppressed (BLIS)	T2N0MX	49	NA	NA
GSM1974664	S3-H134	Basal-Like Immune-Activated (BLIA)	T2N1M0	63	NA	NA
GSM1974663	S3-H133	Luminal-AR (LAR)	T2N0M0	64	20	Post-Menopausal
GSM1974662	S3-H132	Basal-Like Immune-Suppressed (BLIS)	T2N1MX	32	NA	NA
GSM1974661	S3-H131	Basal-Like Immune-Suppressed (BLIS)	T3N0MX	43	NA	NA
GSM1974660	S3-H130	Basal-Like Immune-Activated (BLIA)	T2NXMX	36	28	Pre-Menopausal
GSM1974659	S3-H129	Basal-Like Immune-Activated (BLIA)	T1cN1MX	39	NA	Pre-Menopausal
GSM1974658	S3-H128	Basal-Like Immune-Suppressed (BLIS)	T2N1MX	38	NA	NA
GSM1974657	S3-H127	Basal-Like Immune-Suppressed (BLIS)	T2NXM0	32	22	Pre-Menopausal
GSM1974656	S3-H126	Basal-Like Immune-Activated (BLIA)	T2N0M0	44	25	Pre-Menopausal
GSM1974655	S3-H125	Basal-Like Immune-Suppressed (BLIS)	T2N0M0	62	30	Post-Menopausal
GSM1974654	S3-H124	Mesenchymal (MES)	T2N2MX	43	22	Pre-Menopausal
GSM1974653	S3-H123	Basal-Like Immune-Activated (BLIA)	T2N0M0	57	41	Post-Menopausal
GSM1974652	S3-H122	Luminal-AR (LAR)	T2N0M0	48	21	Pre-Menopausal
GSM1974651	S3-H121	Basal-Like Immune-Suppressed (BLIS)	T2N1MX	45	23	Pre-Menopausal
GSM1974650	S3-H120	Basal-Like Immune-Suppressed (BLIS)	T1N0M0	65	38	Post-Menopausal
GSM1974649	S3-H119	Basal-Like Immune-Suppressed (BLIS)	T2N3M0	70	30	Post-Menopausal
GSM1974648	S3-H118	Mesenchymal (MES)	T2NXM0	67	30	Post-Menopausal
GSM1974647	S3-H117	Basal-Like Immune-Suppressed (BLIS)	T2N0M0	51	31	Pre-Menopausal
GSM1974646	S3-H116	Basal-Like Immune-Activated (BLIA)	T2N3MX	50	NA	NA
GSM1974645	S3-H115	Luminal-AR (LAR)	T2N1M0	38	28	Pre-Menopausal
GSM1974644	S3-H114	Luminal-AR (LAR)	T1N1M0	47	34	Menopausal
GSM1974643	S3-H113	Luminal-AR (LAR)	T2N1M0	57	43	Post-Menopausal
GSM1974642	S3-H111	Basal-Like Immune-Suppressed (BLIS)	T2N0M0	44	32	Post-Menopausal
GSM1974641	S3-H110	Luminal-AR (LAR)	T2N0M0	56	25	Post-Menopausal
GSM1974640	S3-H109	Basal-Like Immune-Activated (BLIA)	T2N1M0	38	27	Pre-Menopausal
GSM1974639	S3-H108	Luminal-AR (LAR)	T1N0M0	48	30	Pre-Menopausal
GSM1974638	S3-H107	Luminal-AR (LAR)	T2N3M0	59	24	Post-Menopausal
GSM1974637	S3-H106	Luminal-AR (LAR)	T1N0M0	55	29	NA

GSM1974636	S3-H105	Basal-Like Immune-Suppressed (BLIS)	T2N0M0	72	24	NA
GSM1974635	S3-H104	Mesenchymal (MES)	T2N0M0	43	26	Pre-Menopausal
GSM1974634	S3-H103	Basal-Like Immune-Suppressed (BLIS)	T2N3M0	70	30	NA
GSM1974633	S3-H102	Basal-Like Immune-Activated (BLIA)	T2NXMX	39	28	Menopausal
GSM1974632	S3-H100	Mesenchymal (MES)	T2NXM0	56	34	Post-Menopausal
GSM1974631	S2-H91B	Basal-Like Immune-Activated (BLIA)	T2N1M0	41	39	Pre-Menopausal
GSM1974630	S2-H90	Basal-Like Immune-Suppressed (BLIS)	T2N3M0	56	25	NA
GSM1974629	S2-H89	Mesenchymal (MES)	T1N0M0	73	30	Post-Menopausal
GSM1974628	S2-H88	Basal-Like Immune-Activated (BLIA)	T2NXM0	67	32	Post-Menopausal
GSM1974627	S2-H85B	Mesenchymal (MES)	T2N0M0	NA	23	NA
GSM1974626	S2-H84	Basal-Like Immune-Activated (BLIA)	T2NXM0	75	32	Post-Menopausal
GSM1974625	S2-H83	Luminal-AR (LAR)	T4bN2M0	26	19	NA
GSM1974624	S2-H82	Basal-Like Immune-Activated (BLIA)	T2NXM0	45	23	Pre-Menopausal
GSM1974623	S2-H81	Basal-Like Immune-Suppressed (BLIS)	T1cN0M0	47	24	Pre-Menopausal
GSM1974622	S2-H80	Luminal-AR (LAR)	T2N0M0	75	25	Post-Menopausal
GSM1974621	S2-H79	Basal-Like Immune-Suppressed (BLIS)	T4bN2M0	36	NA	Pre-Menopausal
GSM1974620	S2-H78	Basal-Like Immune-Activated (BLIA)	T2NXM0	72	28	Post-Menopausal
GSM1974619	S2-H77	Basal-Like Immune-Suppressed (BLIS)	T2N1M0	53	NA	Post-Menopausal
GSM1974618	S2-H76	Basal-Like Immune-Suppressed (BLIS)	T2N0M0	38	25	Pre-Menopausal
GSM1974617	S2-H57C	Basal-Like Immune-Suppressed (BLIS)	TXNXMX	42	32	Pre-Menopausal
GSM1974616	S2-H56B	Luminal-AR (LAR)	T3N0M0	70	16	Post-Menopausal
GSM1974615	S2-H55B	Basal-Like Immune-Activated (BLIA)	T2N1M0	71	28	Post-Menopausal
GSM1974614	S2-H54B	Basal-Like Immune-Activated (BLIA)	T2N2M0	42	31	Pre-Menopausal
GSM1974613	S1-H9	Basal-Like Immune-Activated (BLIA)	T3N1M0	55	30	Post-Menopausal
GSM1974612	S1-H8	Mesenchymal (MES)	T2N1M0	NA	25	Pre-Menopausal
GSM1974611	S1-H73	Basal-Like Immune-Suppressed (BLIS)	TXNXMX	42	29	Pre-Menopausal
GSM1974610	S1-H72B	Luminal-AR (LAR)	T1N0MX	74	29	Post-Menopausal
GSM1974609	S1-H71	Mesenchymal (MES)	T2N0MX	NA	28	Pre-Menopausal
GSM1974608	S1-H70	Mesenchymal (MES)	T2NXM0	64	26	Post-Menopausal
GSM1974607	S1-H7	Basal-Like Immune-Suppressed (BLIS)	T1cN0M0	73	25	Post-Menopausal
GSM1974606	S1-H69	Mesenchymal (MES)	T1NXM0	58	24	Post-Menopausal
GSM1974605	S1-H68	Basal-Like Immune-Activated (BLIA)	T2N1M0	66	33	Post-Menopausal
GSM1974604	S1-H67	Basal-Like Immune-Activated (BLIA)	T2NXM0	48	25	Pre-Menopausal
GSM1974603	S1-H66	Basal-Like Immune-Activated (BLIA)	T2N1M0	65	NA	Post-Menopausal
GSM1974602	S1-H65	Basal-Like Immune-Suppressed (BLIS)	T2N1MO	70	24	Post-Menopausal
GSM1974601	S1-H64	Luminal-AR (LAR)	T2N0M0	48	NA	NA
GSM1974600	S1-H63	Basal-Like Immune-Suppressed (BLIS)	T2N2M0	57	38	Post-Menopausal
GSM1974599	S1-H62	Mesenchymal (MES)	T2N1M0	NA	22	Post-Menopausal
GSM1974598	S1-H61	Basal-Like Immune-Activated (BLIA)	T2NXMX	57	35	Post-Menopausal
GSM1974597	S1-H60	Luminal-AR (LAR)	T4N1M0	49	26	Pre-Menopausal
GSM1974596	S1-H6	Mesenchymal (MES)	T2N0M0	64	36	Post-Menopausal
GSM1974595	S1-H59	Basal-Like Immune-Activated (BLIA)	T2NXMX	49	33	Pre-Menopausal
GSM1974594	S1-H58B	Luminal-AR (LAR)	T2N1M0	67	38	Post-Menopausal
GSM1974593	S1-H53	Basal-Like Immune-Activated (BLIA)	T2N3M0	38	35	Pre-Menopausal

GSM1974592	S1-H52	Basal-Like Immune-Activated (BLIA)	T4bN0M0	35	25	Pre-Menopausal
GSM1974591	S1-H51	Basal-Like Immune-Activated (BLIA)	TXN1M0	75	18	Post-Menopausal
GSM1974590	S1-H50	Mesenchymal (MES)	T2N2M0	51	23	Pre-Menopausal
GSM1974589	S1-H4B	Mesenchymal (MES)	T2N2MX	37	23	Pre-Menopausal
GSM1974588	S1-H49	Basal-Like Immune-Suppressed (BLIS)	T3N0MX	42	NA	Pre-Menopausal
GSM1974587	S1-H48	Mesenchymal (MES)	T2N1M0	71	32	Post-Menopausal
GSM1974586	S1-H47	Basal-Like Immune-Suppressed (BLIS)	T2N1aM0	55	NA	NA
GSM1974585	S1-H46	Basal-Like Immune-Suppressed (BLIS)	T1cN0M0	76	28	NA
GSM1974584	S1-H45	Basal-Like Immune-Suppressed (BLIS)	T2N2M0	45	22	Pre-Menopausal
GSM1974583	S1-H44	Mesenchymal (MES)	T2N1M0	54	34	NA
GSM1974582	S1-H43	Basal-Like Immune-Suppressed (BLIS)	T2N0M1	69	31	Post-Menopausal
GSM1974581	S1-H41	Mesenchymal (MES)	T2N1MX	78	28	Post-Menopausal
GSM1974580	S1-H40	Mesenchymal (MES)	T2N0MX	44	44	NA
GSM1974579	S1-H3B	Mesenchymal (MES)	T4N2MX	55	32	Post-Menopausal
GSM1974578	S1-H38	Mesenchymal (MES)	T2N0M0	NA	29	NA
GSM1974577	S1-H36	Basal-Like Immune-Activated (BLIA)	T2N0M0	69	29	Post-Menopausal
GSM1974576	S1-H35B	Mesenchymal (MES)	T1N0MX	57	38	Post-Menopausal
GSM1974575	S1-H31	Mesenchymal (MES)	T1cNXMX	66	22	Post-Menopausal
GSM1974574	S1-H2B	Luminal-AR (LAR)	T2N0M0	59	26	Post-Menopausal
GSM1974573	S1-H29	Mesenchymal (MES)	T1NXMX	40	22	Pre-menopausal
GSM1974572	S1-H28	Basal-Like Immune-Suppressed (BLIS)	T2N0M0	43	36	Pre-Menopausal
GSM1974571	S1-H27	Mesenchymal (MES)	T2N2MX	65	38	Post-Menopausal
GSM1974570	S1-H22	Mesenchymal (MES)	T1cN0M0	55	31	Post-Menopausal
GSM1974569	S1-H20B	Mesenchymal (MES)	T2N0M0	55	NA	NA
GSM1974568	S1-H19	Mesenchymal (MES)	T1N0M0	41	29	Post-Menopausal
GSM1974567	S1-H14	Basal-Like Immune-Activated (BLIA)	T2N0M0	54	23	Post-Menopausal
GSM1974566	S1-H10	Mesenchymal (MES)	T2N1M0	NA	32	Post-Menopausal

Supplemental table 1. The features of 198 TNBC samples.
